# Prader–Willi syndrome in neonates: twenty cases and review of the literature in Southern China

**DOI:** 10.1186/s12887-016-0662-2

**Published:** 2016-08-09

**Authors:** Ping Wang, Wei Zhou, Weiming Yuan, Longguang Huang, Ning Zhao, Xiaowen Chen

**Affiliations:** 1 The First Affiliated Hospital of Jinan University, No 613, Huangpuda Road, Guangzhou, Guangdong, 510623 China; 2 Department of Neonatology, Guangzhou Women and Children’s Medical Center, No 9, Jinsui Road, Guangzhou, Guangdong, 510623 China

**Keywords:** Prader–Willi syndrome, Intervention, Diagnosis, Infant, Neonatal, Asia

## Abstract

**Background:**

Prader-Willi syndrome is a rare genetic abnormality that can be challenging to diagnose early, but for which early interventions improve prognosis.

**Methods:**

To improve understanding of Prader–Willi syndrome in neonates in Asia, we retrospectively analyzed the clinical records of 20 affected newborns diagnosed in the Department of Neonatology, Guangzhou Women and Children’s Medical Center, Guangzhou, China from January 2007 to December 2014 and performed a review of the relevant literature.

**Results:**

Fourteen boys and six girls presented with hypotonia, poor responsiveness, feeding difficulty, and infrequent, weak crying. Different from western patients, the 20 Asian patients exhibited at least five of the following typical features: prominent forehead, narrow face, almond-shaped eyes, small mouth, downturned mouth, thin upper lip, and micromandible. All 14 boys had a small scrotum, including nine with cryptorchidism. Diagnoses were made with microarray comparative genomic hybridization. All 20 infants required feeding tubes. Fifteen received swallowing training immediately after admission; the period of continuous tube feeding for these patients ranged from 8 to 22 days (mean, 14 ± 5.3 days). For the five patients who did not receive swallowing training, the period of continuous tube feeding ranged from 15 to 35 days (mean, 18 ± 4.3 days). Comprehensive care measures included: giving parents detailed health education and basic information about this disease, teaching skills to promote feeding and prevent suffocation, increasing children’s passive activity, providing nutrition management for normal development, and preventing excessive or inadequate nutrient intake.

**Conclusions:**

Neonates with Prader–Willi syndrome in Asia have hypotonia, poor responsiveness, feeding difficulty, infrequent and weak crying, genital hypoplasia, and characteristic facial features. Recognition of the syndrome in neonates with confirmation by genetic testing is essential, because early diagnosis allows early intervention. Treatment measures including swallowing training can improve prognosis, prevent growth retardation and obesity, and elevate quality of life in individuals with Prader–Willi syndrome.

## Background

Prader–Willi syndrome (PWS) is a complex multisystem abnormality, first reported by Prader and colleagues in 1956. The syndrome is generally caused by an abnormality in the q11-13 region on paternal chromosome 15. The incidence of PWS varies in different countries from 1 in 15,000 to 1 in 25,000, with an average mortality of 3 % [1–3]. The main clinical features of affected individuals include hypotonia, feeding difficulty, developmental delay, short stature, abnormal behavior during the neonatal period, obesity, poor hypothalamic and gonadal development, and characteristic appearance during childhood [4]. There are few reports of the syndrome in Asia. There is currently insufficient understanding of the signs and symptoms of PWS in neonates, and the rate of early diagnosis is not high in Asia. Early diagnosis allows implementation of comprehensive treatment at an early stage, which can improve growth and minimize developmental disorders, improving prognosis. In this study we evaluated the clinical manifestations of PWS in 20 neonates who were treated in the Department of Neonatology, Guangzhou Women and Children’s Medical Center, Guangzhou, China. We also analyzed the relevant literature, and present the features of and comprehensive intervention measures for PWS in neonates in China.

## Methods

### Subjects

All neonates treated in the Department of Neonatology, Guangzhou Women and Children’s Medical Center, Guangzhou, China from January 2007 to December 2014 underwent comprehensive physical examination. Neonates who exhibited prominent forehead, narrow face, almond-shaped eyes, downturned mouth, thin upper lip, micromandible, and/or small scrotum, cryptorchidism and hypotonia were diagnosed with suspected PWS.

### Clinical features

The appearances and characteristics in the 20 neonates with PWS were summarized and analyzed.

### Examination methods

Further examinations were performed in these cases, including cranial magnetic resonance imaging; electroencephalogram; chest X-rays; ultrasonography of the cardiovascular system, urinary system and digestive system; as well as tandem mass spectrometry for congenital metabolic disorders. Affymetrix CytoScan 750K chip was used to detect the variation in patients’ genome. Parental health status was investigated to determine any history of congenital, hereditary, or familial diseases, history of pregnancy and delivery, and pregnancy complications. All 20 patients were diagnosed with PWS based on microarray comparative genomic hybridization within 2 months of birth. Comprehensive interventions were simultaneously instituted, including swallowing training and physical therapy, with regular rehabilitation assessments.

## Results

### General data

Fourteen boys and six girls were hospitalized during the neonatal period. Of the 20 cases, 16 were treated in the Department of Neonatology immediately after birth because of poor responsiveness. Four left the hospital after birth, but presented again because of poor feeding, one at 1 week, and the others at 2~4 weeks of age. Maternal age ranged from 19 to 38 years (mean, 31.5 ± 5.4 years). Paternal age ranged from 21 to 45 years (mean, 34.9 ± 8.2 years). Parents were generally healthy, and with no history of congenital, hereditary, or familial diseases. Mothers of six of the neonates had complained of infrequent fetal movement during pregnancy. Mothers of eight of the neonates had experienced polyhydramnios. None of the mothers had experienced pregnancy complications. Gestational age ranged from 32 to 42 weeks (mean, 38.5 ± 2.7 weeks). Four babies were born at 32~36 weeks (premature birth). The remaining 16 were full term. Five infant was delivered by spontaneous delivery; 15 were delivered by cesarean delivery. Mean birth weight was 2732 ± 265 g. Two patients were full-term small-for-gestational-age infants. Three had mild asphyxia at birth; the remaining 17 did not.

### Features during the neonatal period

**Fig. 1:**
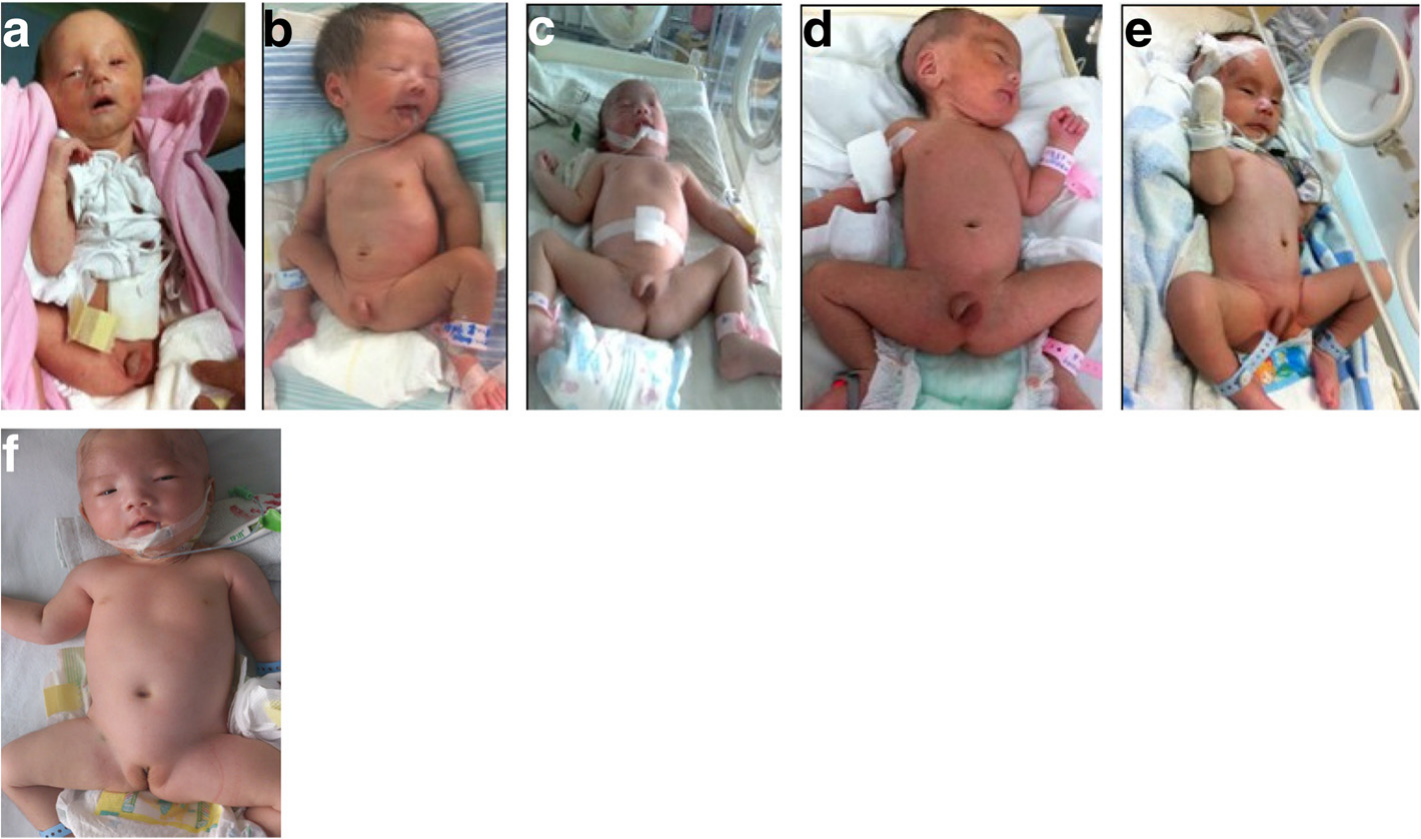
Characteristic sleeping posture and scrotal and vulvar dysplasia in six neonates with Prader–Willi syndrome. **a**–**e** Cases 1–5, respectively. All patients exhibited hypotonia, showing the characteristic sleeping posture of bilateral hip valgus and weakness of limbs. All patients exhibited prominent forehead, almond-shaped eyes and downturned mouth. The boys had a small scrotum. Cases 2, 4, and 5 had cryptorchidism. **f** Case 9, with hypotonia, bilateral hip valgus, and vulvar dysplasia

All 20 neonates presented with hypotonia, poor responsiveness, feeding difficulty, and infrequent and weak crying. Hypotonia resulted in the unusual sleeping posture of bilateral hip valgus and weakness of limbs (Fig. 1). All patients exhibited at least five of the following typical facial features, in order from most to least common, included: prominent forehead, almond-shaped eyes, downturned mouth, narrow face, thin upper lip, micromandible, and small mouth (Table 1, Fig. 1). All 14 boys had small scrota, including nine with cryptorchidism. The genitalia of one girl had the appearance of labia minora.

**Table 1:** Facial features and their prevalence among twenty neonates with Prader–Willi syndrome

Case	Gender	Prominent forehead	Narrow face	Almond-shaped eyes	Small mouth	Downturned mouth	Thin upper lip	Micromandible
1	Male	+	+	+	+	+	+	−
2	Male	+	+	+	−	+	+	+
3	Male	+	+	+	+	+	−	+
4	Male	+	−	+	−	+	+	+
5	Male	+	+	+	−	+	+	+
6	Male	+	+	+	−	+	+	+
7	Female	+	+	+	+	+	+	−
8	Male	+	+	+	+	+	+	+
9	Female	+	+	+	+	+	−	−
10	Female	+	+	+	−	+	+	+
11	Male	+	+	+	+	+	+	−
12	Male	+	+	+	−	+	+	+
13	Male	+	+	+	+	+	−	+
14	Male	+	−	+	−	+	+	+
15	Male	+	+	+	−	+	+	+
16	Male	+	+	+	−	+	+	+
17	Female	+	+	+	+	+	+	−
18	Male	+	+	+	+	+	+	+
19	Female	+	+	+	+	+	−	−
20	Female	+	+	+	+	+	+	−
Proportion		100 %	90 %	100 %	55 %	100 %	80 %	65 %

+: have this feature; −: do not have this feature

### Treatment and examination results

All 20 patients presented with feeding difficulty and required feeding tubes. The period of continuous tube feeding ranged from 8 to 35 days (mean, 19 ± 8.5 days). Fifteen neonates received swallowing training immediately after admission. For these 15, the period of continuous tube feeding ranged from 8 to 22 days (mean, 14 ± 5.3 days). For the five patients who did not receive swallowing training, the period of continuous tube feeding ranged from 15 to 35 days (18 ± 4.3 days). One of the four premature infants received mechanical ventilation, which was removed successfully on the 2nd day after birth. No abnormal signs were detected in any of the patients on cranial magnetic resonance imaging. Electroencephalogram revealed increased slow background activity in 12 cases, mainly continuous low-amplitude electrical activity of 3–10 Hz, mixed with increased low-amplitude fast waves. Normal electroencephalogram waveforms were found in eight cases. Chest X-rays showed consolidation of both lungs in four patients, but no abnormality in the others. Echocardiography revealed patent ductus arteriosus in three cases, and patent foramen ovale in six. Ultrasonography of the urinary and digestive systems was normal in all cases. Tandem mass spectrometry showed a small amount of hydroxy acid in the urine of two patients with congenital metabolic diseases, and a small amount of α-ketoglutarate in one patient, indicating a high catabolic state. No organic acid metabolism was detected in the other 17 cases. Length of hospital stay ranged from 10 to 38 days (mean, 21 ± 8.4 days).

### Genetic analysis

**Fig. 2:**
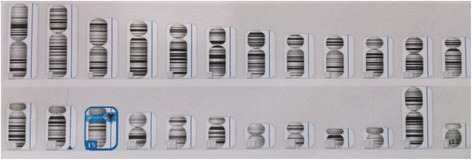
Genotype in case 4: 46,XY. arr14q32.33(106,272,897-106,927,569)×3. arr15q11.2q13.1(23,290,787-28,540,345)×1. Approximately 655-kb repeat in the q32.33 106,272,897-106,927,569 regions on chromosome 14; this repeat is polymorphic. Approximately 5.3-Mb deletion in the q11.2-q13.1 23, 290, 787-28, 540, 345 regions on chromosome 15; this deletion is pathogenic

Genetic analysis was performed with microarray comparative genomic hybridization in all 20 neonates. There were microdeletions in the paternal 15q11-13 region in 16 cases (Fig. 2), and maternal homologous diploid in four cases.

## Discussion

PWS is a complex disease affecting multiple body systems, with variable clinical features in different growth stages. Holm et al. [5] proposed the clinical diagnostic criteria for PWS in 1993. These include a series of symptoms from the perinatal period to adolescence; however, early diagnosis in neonates is difficult based on these criteria. A diagnosis of PWS is reliable at about 2.5 years of age [6], based on a limited number of clinical studies in Asia. Most cases are reported after the neonatal period. In seven studies, patient age at final diagnosis ranged from 1 to 20 years [7–13]; some of these patients presented because of delayed sexual development. Delays in diagnosis and treatment affect patient prognosis and quality of life. Several recent Asian studies have focused on PWS. However, most have explored the genetic diagnosis of the syndrome, and few have investigated manifestations and interventions during the neonatal period. Early diagnosis and early intervention greatly improve the prognosis of individuals with PWS. [6] Therefore, it is important to summarize the neonatal signs and symptoms of the syndrome in Asian, to perform gene detection for suspected cases, and to educate physicians, especially neonatologists, about early diagnosis and intervention.

This study demonstrated that the major clinical features of PWS in China include hypotonia, poor responsiveness, feeding difficulty, and infrequent crying; of these, hypotonia and feeding difficulty are consistent with the findings of previous studies [14–16]. Because of hypotonia, both hips are slightly valgus, resulting in a characteristic sleeping posture in neonates with PWS. Less fetal movement and polyhydramnios are common during pregnancy in women carrying an infant with PWS, and are also associated with hypotonia and feeding difficulty [17]. In the present study, all male patients had a small scrotum, indicating that genital hypoplasia can be considered a reference for diagnosis of PWS in the neonatal period. This series included only six female patients, three of whom had vulvar dysplasia, so we cannot draw conclusions about female genital characteristics associated with the syndrome. A large sample study is needed.

For many years there has been controversy about the characteristic facial features of individuals with PWS. Gunay-Aygun et al. [4] retrospectively analyzed 90 cases of PWS, and confirmed that detection of the syndrome based on particular facial features had a sensitivity of only 49 %. All patients in this study exhibited at least five of the typical facial features, which is consistent with the results of a previous clinical study of five neonates with PWS [18]. These findings suggest that craniofacial features have a high specificity in neonates with PWS in Asia, and are thus very important in diagnosing the syndrome during the neonatal period. The difference of craniofacial features among studies may depend on the different races. Most previous studies [4, 5] were retrospective studies in children or adults, and did not exclude atypical craniofacial features resulting from childhood or adolescent obesity.

Wharton et al. [19] found that approximately 23 % of patients with PWS experienced birth asphyxia; however, Trifiro et al. [20] reported that patients did not suffer from perinatal asphyxia or severe respiratory distress immediately after birth. Three patients in the present study experienced mild birth asphyxia, a proportion similar to that in the report of Wharton et al. All had decreased muscle tone, color, and responsiveness, which are inherent characteristics of neonates with PWS, and have little association with intrauterine or peripartum hypoxia. None of the patients in this study experienced severe respiratory distress. Cranial computed tomography did not reveal signs of hypoxic brain damage. Therefore, we conclude that asphyxia and hypoxia at birth are not very common among neonates with PWS. A previous study demonstrated that the incidence of congenital birth defects such as congenital heart disease and spinal deformity was higher among individuals with PWS and should attract the attention of physicians [21].

Feeding difficulty is common among neonates with PWS. The proportion of neonates with PWS requiring a feeding tube is as high as 90–100 % in various studies. A previous study demonstrated that the average period of feeding tube use in neonates with PWS was 15 days (range, 0–60 days) [6]. All 20 patients in this study required a feeding tube. It reveals that the swallowing capability of PWS neonates is weak regardless of different races. The period of tube feeding was shorter for neonates who received swallowing training than for those who did not. However, the present study had a small sample size; the effect of swallowing training should be confirmed in a larger study. A correlation has been reported between delayed language development and the duration of tube feeding in neonates with PWS [6]. Thus, the period of tube feeding should be shortened, and swallowing training can be conducted at an early stage. In our present study, the patients received nasogastric tube feeding for a median duration of 18 ± 4.3 days. The duration of gastric tube feeding was similar with N. Bachere’s cohort study. The duration of gastric tube feeding tends to decrease, compared with historical cohort, with the younger neonates being less tube-fed than the older ones. This finding is of major importance, as the specific speech and language problems observed in previous patients could be worsened by a long duration of tube feeding. In the future, it would be better to try to avoid gastric feeding by teaching parents how to encourage and optimize suckling.

There is no specific treatment for PWS, so combination therapy is generally implemented. A previous study found a positive therapeutic effect associated with shortening the period of tube feeding, increasing growth in height, controlling abnormal weight gain, and promoting normal growth and development [6]. Parents need to receive detailed health education, including basic information about this disease. They also require instruction on promoting feeding and preventing suffocation, increasing children’s passive activity, providing nutrition management for normal development, and preventing excessive or inadequate nutrient intake. Exercise therapy was performed twice weekly in the present study to improve muscle tone and motor development. From the time of diagnosis, many professionals are involved in the care of children with PWS, including speech therapists, nutritionists, neurologists, psychiatrists, psychologists, and orthopedic surgeons. Previous studies have shown that the incidence of systolic dysfunction is higher in infants with PWS than in unaffected children. Thus, infants with PWS should have cardiovascular, nutritional, endocrine, and physical therapy visits every 3 months during the first year of life. Rechecks can occur every 6 months after the first year. Early diagnosis and intervention allow implementation of reasonable nutrition programs to prevent obesity, improve learning, and assist the development of speech [22, 23]. Regular and timely use of growth hormone (GH) after early diagnosis not only promotes growth and improves fat utilization, but also possibly elevates cognitive level [24, 25]. GH treatment is beneficial for children with PWS. It improves linear growth, body composition, physical strength and agility [26–28]. Fat utilization and resting energy expenditure are increased by GH treatment, the latter partially explained by an increment in lean body mass [29–31]. According to observational data, GH treatment is usually initiated at a mean age of 7 years, as reported by Takeda et al. [32]. Increasingly, GH treatment is initiated earlier [33–35]. Published data support benefits of GH treatment when started between 4 and 6 months of age [36, 37], but some experts are currently treating from as early as 3 months. No consensus was reached on age of GH start, although all agreed to the benefits of treating before the onset of obesity, which often begins by 2 years of age. All of these should be considered by physicians in Asia, and correlated information should be sent to the patients’ parents as early as possible.

## Conclusions

The early clinical manifestations of PWS are not specific, and the disorder is easily confused with other nervous system diseases, increasing the diagnostic challenge. But it still has some special features in Asian neonates. If unexplained hypotonia and feeding difficulty appear during the neonatal period, clinicians should suspect PWS. Infants who exhibit the typical craniofacial features of PWS should undergo genetic testing. When PWS is diagnosed, therapy should be instituted as early as possible. To optimize the prognosis for these patients, the period of tube feeding should be shortened and parents should be provided with health education.

## Abbreviations

PWS, Prader–Willi syndrome; GH, growth hormone
